# Lectures on the Theory and Practice of Physic

**Published:** 1845-01

**Authors:** 


Art. VII.—*Lectures on the Theory and Practice of Physic.* By John Bell, M.D., Fellow of the College of Physicians of Philadelphia; Corresponding Secretary of the Philadelphia Medical College; Member of the American Philosophical Society, and of the Georgofili Society of Florence, etc., etc.; and by William Stokes, M.D., Lecturer at the Medical School, Park Street, Dublin; Physician to the Meath Hospital, etc., etc. Third edition, enlarged and improved. In two volumes. Philadelphia. Ed. Barrington & Geo. D. Haswell. 1845. 8vo. p.p. 1518.

Reserving for our next number a more extended notice of these valuable Lectures, we take great pleasure in thus early introducing this new edition to our readers. The joint labors of Dr. Bell and Dr. Stokes have been eminently successful. Few medical works issued from the American press, within the same period, have had more currency, or been more highly approved by the profession. This edition of the Lectures is marked by substantial improvements, which will enhance the value of the work to the practitioner. Dr. Bell is just the man to keep his book up to the present state of medical science, and his readers have the comfortable assurance that they have before them all the light of recent discovery. Mr. James Maxwell has just received this work.

